# Long noncoding RNA KCNMA1-AS2 regulates the function of colorectal cancer cells and sponges miR-1227-5p

**DOI:** 10.1186/s12885-024-12608-9

**Published:** 2024-07-18

**Authors:** Xinzhi Miao, Fang Wang, Muhammad Amir Yunus, Ida Shazrina Ismail, Tianyun Wang

**Affiliations:** 1 The School of Medical Humanities, Xinxiang Medical University, Xinxiang, Henan province 453003 China; 2 Department of Biomedical Sciences, Advanced Medical and Dental Institute, Universiti Sains Malaysia, Bertam, Kepala Batas, Penang 13200 Malaysia; 3 Henan International Joint Laboratory of Recombinant Pharmaceutical Protein Expression System, Xinxiang Medical University, Xinxiang, Henan province 453003 China; 4 Department of Biochemistry and Molecular Biology, Xinxiang Medical University, Xinxiang, Henan province 453003 China

**Keywords:** KCNMA1-AS2, Biomarker, Colorectal cancer, miR-1227-5p, Long noncoding RNA

## Abstract

**Background:**

Many long noncoding RNAs (lncRNAs) with altered expression significantly influence colorectal cancer (CRC) progression and behavior. The functions of many lncRNAs in CRC are not clear yet. This study aimed to discover novel lncRNA entities and comprehensively examine and validate their roles and underlying molecular mechanisms in CRC.

**Methods:**

Tissue samples, both tumourous and non-tumourous, from three CRC patients were submitted for sequencing. Following expression validation in samples from ten patients and four CRC cell lines. The lncRNA KCNMA1-AS2 was synthesized by In-vitro transcription RNA synthesis and the lncRNA was directly transfected into CRC cell lines to overexpress. Functional assays including MTT proliferation assay, Annexin-V/propidium iodide apoptosis assay, wound healing migration assay and cell cycle assays were performed to evaluate the effect of overexpression of KCNMA1-AS2. Furthermore, the binding of KCNMA1-AS2 to miR-1227-5p was confirmed using dual luciferase reporter assays and qPCR analyses. Subsequent bioinformatics analyses identified 58 potential downstream targets of miR-1227-5p across three databases.

**Results:**

In this study, we identified the lncRNA KCNMA1-AS2, the expression of which was down-regulated consistently in cancer tissues and CRC cell lines compared to non-cancerous tissues. The overexpression of lncRNA KCNMA1-AS2 led to significant reduction in CRC cell proliferation and migration, increase in cell apoptosis, and more cells arrested in S phase. Additionally, the interaction between KCNMA1-AS2 and miR-1227-5p was confirmed through dual luciferase reporter assay and qPCR analysis. It is also putatively predicted that MTHFR and ST8SIA2 may be linked to CRC based on bioinformatics analyses.

**Conclusions:**

LncRNA KCNMA1-AS2 exhibited distinct gene expression patterns in both CRC tissue and cell lines, impacting various cellular functions while also acting as a sponge for miR-1227-5p.The findings spotlight lncRNA KCNMA1-AS2 as a potential marker for diagnosis and treatment of CRC.

**Supplementary Information:**

The online version contains supplementary material available at 10.1186/s12885-024-12608-9.

## Introduction

Colorectal cancer (CRC) is a malignant condition originating from the genetic and epigenetic changes in the colon epithelial mucosa, affected by both environmental and hereditary factors. Age, geography, gender, lifestyle, habits, pollutants, and genetics contribute to the incidence, progression, and treatment response [1]. More than 1.9 million new cases and 935,000 deaths of CRC were reported worldwide in 2020, ranking 3^rd^ in incidence and 4^th^ in mortality among all cancer types [2]. CRC is the most common cancer type among men and the second most common type of cancer among women in Malaysia [3], as well as the third most frequently diagnosed type of cancer in China [4]. Early signs and symptoms of CRC are often understated, leading to delayed diagnosis and treatment. Advanced metastasis is found in approximately 20% of the patients at initial diagnosis. Untreated metastatic CRC has a stark 5-year survival rate of just 3% [5].

Long noncoding RNA (lncRNA) is a category of noncoding RNA molecules, exceeding 200 nucleotides in length, primarily transcribed by RNA polymerase II. LncRNAs molecules are not structured to encode proteins. However, molecular biology researches have highlighted the pivotal role of lncRNAs in tumor regulation. LncRNAs may be key markers in predicting diagnosis and treatment response of CRC [6, 7]. LncRNAs may interplay with DNA, mRNA, noncoding RNA, and proteins to exert biological effects [8]. LncRNAs also act as microRNA (miRNA) sponges in the competitive endogenous RNA (ceRNA) regulatory network (i.e., lncRNA-miRNA-mRNA system), inhibiting miRNA expression and thus regulating downstream genes [9]. As an example, a prior investigation demonstrated that Linc00938 could directly bind to hsa-miR-30c-5p, potentially regulating LRRK2 expression through the miR-30c-5p sponge mechanism. This finding offers fresh insights into the ceRNA network involving lncRNA, miRNA, and mRNA in the regulation of disease genes [10]. Alterations in lncRNA expression can induce significant functional shifts involving cell proliferation, apoptosis, migration, and invasion of CRC cells [11].

Limited awareness of CRC often leads to late-stage diagnosis, underscoring the urgent need for new biomarkers to enable early detection. LncRNAs have emerged as a focus in CRC research in recent years. These molecules are intricately linked to the development and progression of tumors [12–15]. A number of lncRNAs have been identified. However, the functions of many lncRNAs are not clear yet. This study aims to explore the relevant new lncRNAs in CRC and characterize their potential roles in early detection, diagnosis, and treatment of CRC.

During this study, KCNMA1-AS2 was identified as a lncRNA that was down-regulated in CRC tissues and cell lines. It was found to exert anti-proliferative effect, promoting apoptosis, inhibiting migration, and inducing cell cycle arrest in CRC cells. Confirmation of the binding between KCNMA1-AS2 and miR-1227-5p, along with bioinformatics analyses, unveiled 58 potential downstream targets of miR-1227-5p, including 10 identified as cancer-related. Notably, MTHFR and ST8SIA2 were implicated in CRC. KCNMA1-AS2 exhibits promise as a diagnostic and therapeutic biomarker for CRC.

## Materials and methods

### Tissue collection and lncRNA expression profiling

Patients who were diagnosed at different clinical stages of CRC at the First Affiliated Hospital of Xinxiang Medical University in China were included in this study. They did not have other underlying diseases or receive any anti-cancer treatment prior to study entry. Tissue samples, both tumor and non-tumor, were obtained from the enrolled CRC patients. Non-tumorous samples were collected from locations 5 cm away from the tumor-affected areas. After extraction, tissues were immediately snap-frozen in liquid nitrogen and stored at -80 °C until analysis. The experiments were undertaken with the understanding and written consent of each subject. Both tumorous and adjacent non-tumorous tissue specimens were sent to Personal Biotechnology Co., Ltd (Shanghai, China) for Next-Generation Sequencing. This sequencing was done on the Illumina HiSeq platform, employing a paired-end sequencing approach. Stringtie v1.3.3 software was employed for abundance analysis of known genes or transcripts, utilizing alignment results from Tophat (http://tophat.cbcb.umd.edu/). LncRNA filtering involved the assembly outcomes of two software applications and structural characteristics, with criteria including length ≥ 200 bp, a minimum of two exons, minimum read coverage ≥ 3, exclusion of transcripts identical or similar to known ones, and identification of newly candidate lncRNAs using Class_Code information from gffcompare results (http://cufflinks.cbcb.umd.edu/manual.html#class_codes). The DESeq performed differential expression analysis of lncRNAs at the transcript level. A lncRNA was identified as significantly differentially expressed lncRNA if *p* < 0.05 and |log_2_(Fold Change) | > 1.

### KCNMA1-AS2 expression validation in CRC tissue samples and cell lines by quantitative real-time polymerase chain reaction (qPCR)

Total RNA was extracted from CRC tissues, normal human colon mucosal epithelial cell line NCM460 and CRC cell lines (HCT116, Caco-2, HT29, and SW1463) using TRIzol™ Reagent (Thermo Fisher Scientific, Massachusetts, USA). The RNA from normal tissues and the NCM460 served as controls. RNA integrity was verified through denaturing-agarose gel electrophoresis and nanodrop spectrophotometer (Thermo Fisher Scientific, Massachusetts, USA). All cell lines were cultivated in specific complete media: NCM460, SW1463, and HT-29 in RPMI 1460; Caco-2 in DMEM with high glucose; and HCT116 in MEM. Each medium (Thermo Fisher Scientific, Massachusetts, USA) was supplemented with 10% FBS and 1% Pen-Strep, and cells were maintained at 37 °C in 5% CO_2_. The SuperScript™ IV First-Strand Synthesis System (Thermo Fisher Scientific, Massachusetts, USA) facilitated cDNA reverse transcription. The qPCR assay was used with the SensiFAST™ SYBR^®^ Hi-ROX Kit (Bioline, London, UK) to quantify and confirm KCNMA1-AS2’s expression levels in the samples. Specific primers were used for amplification of KCNMA1-AS2 and GAPDH (Table S1). The qPCR reactions were performed via StepOnePlus™ Real-Time PCR System (Thermo Fisher Scientific, Massachusetts, USA). The 2^−ΔΔCt^ technique was adopted to express the differential lncRNA expression levels.

### Establishing KCNMA1-AS2 overexpression CRC cell line model systems

A gBlock gene fragment dsDNA (consisting of T7 promoter and lncRNA KCNMA1-AS2) was procured from Apical Scientific (Selangor, Malaysia). The full sequence was then amplified via PCR using specific primers and the Q5 High-Fidelity 2X Master Mix (New England Biolabs, Massachusetts, USA). The resulting PCR product underwent purification using 0.3 M NaAc and 70% ethanol to obtain a pure DNA template. The desired lncRNA was synthesized from this template using the HiScribe T7 Quick High Yield RNA Synthesis Kit (New England Biolabs, Massachusetts, USA), followed by purification with DNase I & LiCl. The concentration and purity of the synthesized lncRNA were assessed using nanodrop. The prepared RNA was subsequently stored at -80 °C until assay.

For the transfection procedure, cells were prepared a day in advance by seeding 3 × 10^5^ cells into each well of a 12-well plate with 1 mL of antibiotic-free medium, ensuring approximately 50% confluency on the day of transfection. Transfection was carried out using 10 ng of either the lncRNA or a blank control, employing the Lipofectamine™ 2000 Transfection Reagent (Thermo Fisher Scientific, Massachusetts, USA). Post-transfection, cells were maintained at 37 °C in a CO_2_ incubator for either 24–48 h intervals. At the end of each interval, cells were collected, and their RNA was isolated using TRIzol reagent. The efficiency of lncRNA overexpression was assessed by qPCR.

### Functional assays

#### Proliferation assay

Cell proliferation post-lncRNA overexpression in CRC cells was assessed using the 3-[4,5-dimethylthiazol-2-yl]-2,5 diphenyl tetrazolium bromide (MTT) assay. Cells were seeded in a 96-well plate at a density of 1 × 10^4^ cells per well and incubated in 5% CO_2_, 95% air humidified incubator at 37 ℃ overnight. The subsequent day, cells were transfected with purified lncRNA. Cells without treatment acted as the negative control (NC) for the overexpression group. After intervals of 0, 24, 48, and 72 h, the medium in the wells was discarded. Each well was then treated with an MTT solution comprising 100 µL of fresh medium and 10 µL of MTT (Thermo Fisher Scientific, Massachusetts, USA). The cells were then incubated for an additional 4 h at 37℃. This solution was subsequently removed, and 100 µL dimethyl sulfoxide (Thermo Fisher Scientific, Massachusetts, USA) was added to each well. The plate was incubated for 10 min, shielded with tinfoil, and gently agitated before reading. Absorbance values were read at 540 nm.

#### Annexin-V/propidium iodide (PI) apoptosis assay

The effects of overexpressing KCNMA1-AS2 on cell apoptosis were assessed using the Annexin V-FITC apoptosis detection kit (Beyotime Biotechnology, Shanghai, China). The culture medium from the cells was transferred to a fresh centrifuge tube. After rinsing the cells in the 6-well plate with PBS once, they were treated with 500 µL of trypsin-EDTA for detachment. Once trypsinized, the trypsin was discarded and the reserved cell culture medium was reintroduced. Cells were then carefully re-suspended and shifted to the centrifuge tube. The gathered cells were centrifuged at 1000 x g for 5 min. The cell pellet was obtained after discarding the supernatant. The cells were subsequently rinsed and re-suspended in PBS, followed by counting. From this, 5 × 10^4^ cells were taken, centrifuged and the supernatant was removed. The cell pellet was then gently resuspended in 195 µL of Annexin V-FITC binding solution. 5 µL of Annexin V-FITC was introduced to this suspension and blended. The mixture was left to incubate at room temperature for 15 min. Then, 10 µL of PI staining solution was incorporated and gently mixed. Additional 300 µL of PBS was added, and the cells were shielded from light and left to incubate for 15 min, subsequently being kept on ice in darkness. After collection and a cold PBS wash, the cell samples were analyzed with the BD FACSCanto™ Flow Cytometry System (BD Biosciences, California, USA). The collected data were interpreted using the Flowjo v.10.8.1 software.

#### Cell cycle assay

The effect of overexpressing KCNMA1-AS2 on cell cycle was assessed through a cell cycle assay to determine any potential phase changes or disruptions. At intervals of 24, 48, and 72 h post-transfection, the culture medium was transferred to a 4 mL centrifuge tube. Cells from the 6-well plate were detached using trypsin, harvested, and then combined with the previously collected medium. This was then centrifuged at 1000 x g for 5 min to gather the cells. The cells were washed twice using 1 mL of pre-cooled PBS. They were gently resuspended in 1 mL of cold ethanol and incubated at 4℃ for 2 h to fix the cells. Post incubation, the cells were centrifuged again at 1000 x g for 5 min, the supernatant was removed, and the cells were washed with 1 mL of chilled PBS to clear away residual ethanol. The PI staining solution was prepared according to the manufacturer’s instructions (Beyotime Biotechnology, Shanghai, China). Each cell sample was treated with 0.5 mL of the PI staining solution, resuspended, and then kept in darkness at 37℃ for 30 min. After staining, the cells were transferred to flow cytometry tubes and maintained on ice in darkness. The cell cycle distribution was analyzed using the BD FACSCanto™ Flow Cytometry System, and data interpretation was done using ModFit LT v.5.0 software.

#### Cell migration assay

The influence of KCNMA1-AS2 overexpression on the migration ability of CRC cells was examined using a wound healing assay. Prior to transfection, HCT116 and SW1463 cells were plated on a 6-well plate with medium supplemented with 10% FBS and incubated at 37℃. On the day of transfection, once cells achieved approximately 90% confluence, they were transfected with 20 ng of lncRNA per well using Lipofectamine 2000 and then incubated for additional 6 h. Following this incubation, a clear linear wound was made in the cell layer using a sterile 200 µL micropipette tip. The debris was cleared by washing the cells with PBS, and then the medium was switched to RPMI1640 containing 2% FBS. The commencement of the wound was set as the 0-hour mark. Cells were monitored using a Nikon Inverted Microscope Eclipse Ti-S, and images were captured at intervals: 0, 24, 48, and 72 h post-wounding. The wound’s width was quantified using ImageJ v1.51 software.

### Potential association of lnRNA with miRNA and target genes

#### Vector construction

The potential target miRNA for lncRNA KCNMA1-AS2 was predicted using DIANA tools lncBase v.2 [16]. The target miRNAs were selected based on the overall score greater than 0.9 and those which have been described or reported in other publications. The potential binding site on the lncRNA was labeled as wild-type (WT). Its corresponding base was substituted with its complementary base, as a mutant (MUT). The relationship between the lncRNA and the target miRNA was examined using the dual luciferase reporter assay, employing the psiCHECK-2 plasmid (Promega, Madison, USA). Both WT and MUT dsDNA PCR products of KCNMA1-AS2 were produced by General Biosystems Co., Ltd (Anhui, China). Dry powder products (500 ng) were reconstituted in 20 µL H_2_O, yielding a concentration of 25 ng/µL. These were preserved at -20 ℃ for future assays.

#### Restriction enzyme digestion, gel extraction and ligation

The integration of KCNMA1-AS2 WT or MUT into the vector was facilitated using the XhoI/NotI restriction enzyme sites (Takara, Shiga, Japan). Both the dsDNA PCR products and the psiCHECK-2 vector were subjected to digestion with XhoI and NotI enzymes at 37 ℃ for 5.5 h. Subsequently, the digested samples were run on 1% agarose gel at 70 V for 30 min. The desired band was visualized under UV light, excised, and then purified using the Gel Extraction Kit (Cwbio, Jiangsu, China). These digested PCR fragments were then inserted downstream of the Renilla luciferase gene in the psiCHECK-2 vector with the aid of T4 DNA Ligase (Takara, Shiga, Japan), resulting in psiCHECK-2 WT and psiCHECK-2 MUT constructs.

#### Transformation, cloning and plasmid extraction

The ligation mixture (5 µL) was introduced into 65 µL of DH5α competent cells (Thermo Fisher Scientific, Massachusetts, USA) and kept on ice for 30 min. Subsequently, the cells were exposed to a 42 °C water bath for a precise duration of 90 s for heat-shock, followed by a cooling period on ice for 5 min. After adding 500 µL of LB medium, the mixture underwent agitation at a speed of 225 rpm for an hour at 37 °C. A 100 µL aliquot of the cell suspension was then plated onto LB agar supplemented with ampicillin. The plate was inverted and incubated overnight at 37 °C. By the following afternoon, an individual colony was selected and inoculated into 5 mL of LB medium supplemented with 5 µL of ampicillin and then incubated with shaking overnight at 37 °C. The next day, the recombinant plasmid was isolated using the EndoFree Plasmid Mini Kit (Cwbio, Jiangsu, China). The isolated plasmid was preserved at -20 °C. For validation, the plasmid was forwarded to Sangon Biotech Co., Ltd. (Shanghai, China) to ensure both its structural integrity and sequencing accuracy.

#### Dual luciferase reporter assay

GenePharma company (Shanghai, China) synthesized both the miRNA mimics and the NC. In 96-well plates, HCT116 and SW1463 cells were jointly transfected with either the reconstructed psiCHECK2-WT or psiCHECK2-MUT (0.2 µg/well), and the miR-1227-5p mimics or NC at a concentration of 13 pmol, using Lipofectamine 2000. The cells were collected after 48 h incubation. Luciferase assays were subsequently conducted using the Dual Luciferase Reporter Gene Assay Kit (Beyotime Biotechnology, Shanghai, China). A SpectraMax i3 microplate reader (Molecular Devices, California, USA) was employed to measure the luciferase activity. For normalization, the data from the luciferase were adjusted by determining the Renilla luciferase to firefly luciferase ratio. This procedure was independently replicated thrice using different samples.

#### miRNA qPCR

The assay of miRNA qPCR was carried out to assess miR-1227-5p expression in CRC cell lines following KCNMA1-AS2 upregulation. RNA was harvested from HCT116 and SW1463 cells 24 h and 48 h post-transfection with KCNMA1-AS2. For comparison, RNA was also extracted from a control group of untransfected cells. The RevertAid First Strand cDNA Synthesis Kit (Thermo Fisher Scientific, Massachusetts, USA) was used for miRNA reverse transcription. The primer sequences were listed in Table S2. The qPCR procedure was executed using the StepOne™ Plus system. The 2^−ΔΔCt^ technique was used to depict the variations in target lncRNA expression levels between the test group and control group. The entire experiment was conducted three times to ensure consistent results.

#### miRNA target gene prediction

The miRNA-mRNA network was predicted using miRDB [17], DIANA microT-CDS [18], and TargetScan version 7.2 [19]. In these tools, the species ‘Homo sapiens’ was chosen, and the target of miR-1227-5p was predicted. Subsequently, all prediction target result from these tools were exported. Then the different filtering criteria were applied to each database: targetScan (Cumulative weighted context + + score ≤ -0.3), miRDB (score ≥ 60), and DIANA microT-CDS (miTG score ≥ 0.8). Genes that aligned with the previously mentioned selection criteria and were consistently identified across all three databases were chosen as common potential targets of miR-1227-5p. Subsequent bioinformatic evaluation, encompassing pathways and associated diseases, were conducted on these common potential targets via the KOBAS database [20]. Gene symbols were utilized to input all common potential targets, and bioinformatic analysis was conducted specifically under the Homo sapiens species. Pathway and disease information were summarized and filtered to identify target genes associated with cancer, with a particular focus on those related to CRC. This approach aimed to pinpoint potential target genes linked to CRC.

### Statistical analysis

Statistical evaluation was performed using GraphPad Prism software v.9.0.0. Results are expressed as mean ± SD. The Shapiro-Wilk test was utilized to check the normality of data distribution. Student’s *t*-test was used to compare between two groups for the data with a normal distribution. Mann-Whitney test was used for the abnormally distributed data. One-way analysis of variance (ANOVA) was used for comparison among multiple groups. The data with normal distribution were subsequently assessed using Brown-Forsythe and Welch ANOVA tests, followed by either Tukey’s or Dunnett’s T3 post hoc tests. For the data of abnormal distribution, Kruskal-Wallis test combined with Dunn’s post hoc correction was employed. Two-way ANOVA and Šídák’s post hoc test were used to analyze the data of functional assays. *p* < 0.05 was considered statistically significant.

## Results

### KCNMA1-AS2 was down-regulated in the CRC tissues and cells

**Fig. 1:**
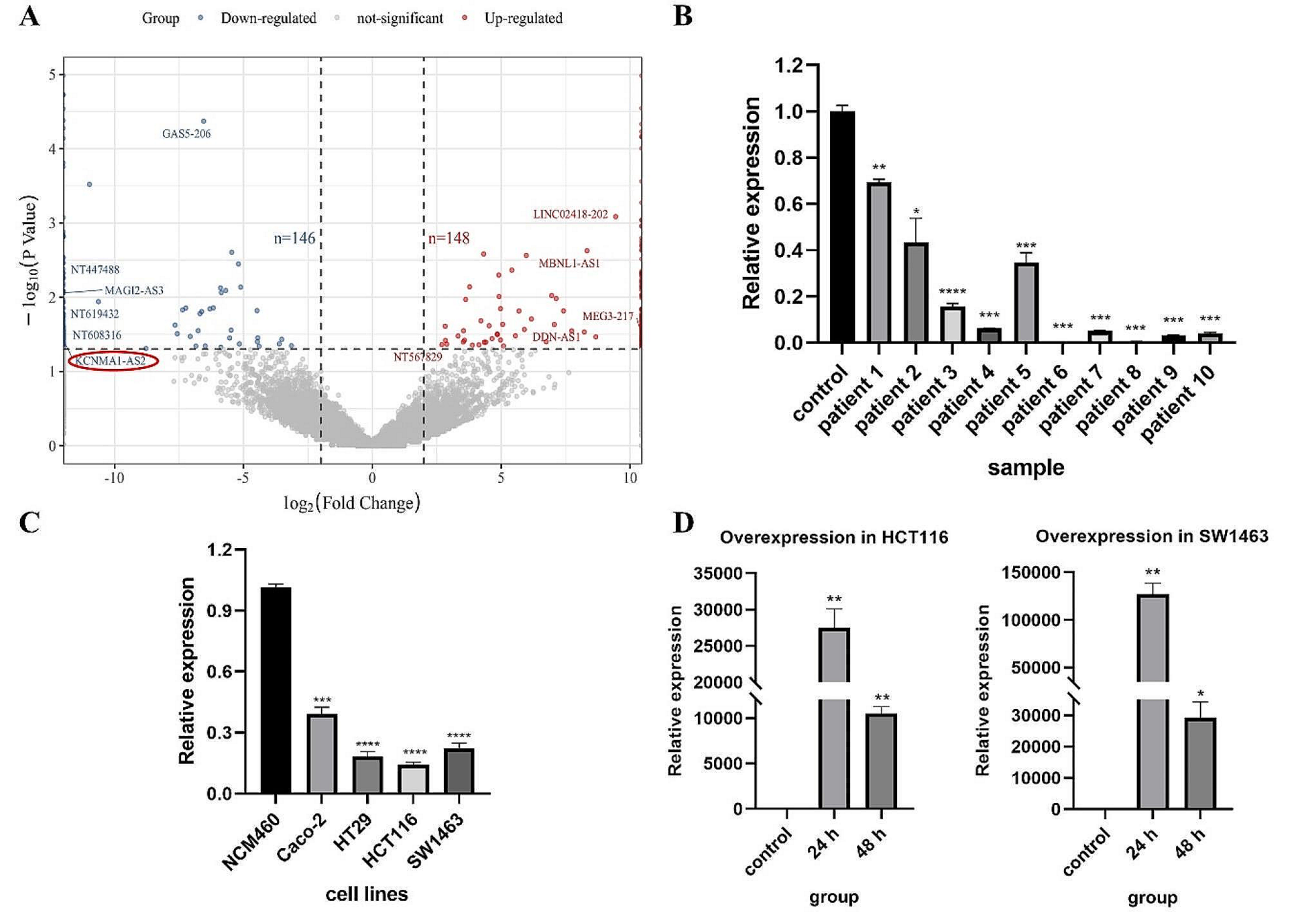
KCNMA1-AS2 was down-regulated in the CRC tissues and cells. (**A**) The volcano plot illustrated the differential expression of lncRNAs. Within this symmetrically distributed plot, lncRNA KCNMA1-AS2, positioned on the left side, signified its downregulation in tumor tissues relative to non-tumor tissues. (**B**) qPCR assessment of KCNMA1-AS2’s relative expression in tumor tissues versus non-tumor tissues. Reduced expression of KCNMA1-AS2 was noted consistently across all of the tumor samples from 10 patients. (**C**) Comparative analysis of lncRNA KCNMA1-AS2’s relative expression in Caco-2, HT29, HCT116, and SW1463 cells against NCM460 cells, using qPCR. KCNMA1-AS2’s expression was reduced significantly across the four CRC cell lines compared with the control NCM460 cells. (**D**) Expression of lncRNA KCNMA1-AS2 in HCT116 and SW1463 cells at 24 h and 48 h post-transfection compared with the untransfected control by qPCR. A significant uptick in expression was recorded in both cell lines (*p < 0.05, **p < 0.01, ***p < 0.001, ****p < 0.0001)

Tumor and non-tumor tissue specimens were obtained from three patients, followed by sequencing analysis to identify the lncRNAs showing differential expression patterns. The sequencing procedure identified a total of 22,333 lncRNAs. Significant expression disparities were observed in the tumor tissues: 148 lncRNAs were up-regulated, while 146 were down-regulated significantly (Fig. 1A). Specifically, the expression of lncRNA KCNMA1-AS2 was reduced significantly in tumor tissues (*p* = 0.03697, log_2_(Fold Change) = -infinity). To validate the findings derived from sequencing, a qPCR analysis was performed. The levels of KCNMA1-AS2 expression in tumor tissues were compared to those in non-tumor counterparts from CRC patients (Fig. 1B). Utilizing qPCR validation with tissue samples from 10 patients, it was observed that the expression of KCNMA1-AS2 in tumor tissue was consistently down-regulated compared to non-tumor tissue in each patient. The *p*-values varied, with patient 2 showing *p* < 0.05, patient 1 showing *p* < 0.01, and the remaining 8 patients exhibiting *p* values less than 0.001. This confirmed a consistent reduction in KCNMA1-AS2 expression across all 10 tumor specimens. Furthermore, in an extended qPCR validation of lncRNA expression, significant down-regulation of KCNMA1-AS2 was found in all of the CRC cell lines when compared with the control NCM460 cells (Fig. 1C).

To achieve overexpression of lncRNA KCNMA1-AS2, a synthesized in-vitro lncRNA was introduced directly into two CRC cell lines: HCT116 and SW1463. Cells that did not undergo transfection served as the control group. Relative expression levels were assessed using qPCR at intervals of 24 h and 48 h post-transfection. As illustrated in Fig. 1D, a marked elevation in expression was observed in both cell lines.

### Overexpression of KCNMA1-AS2 inhibited proliferation and promoted apoptosis of CRC cells

**Fig. 2:**
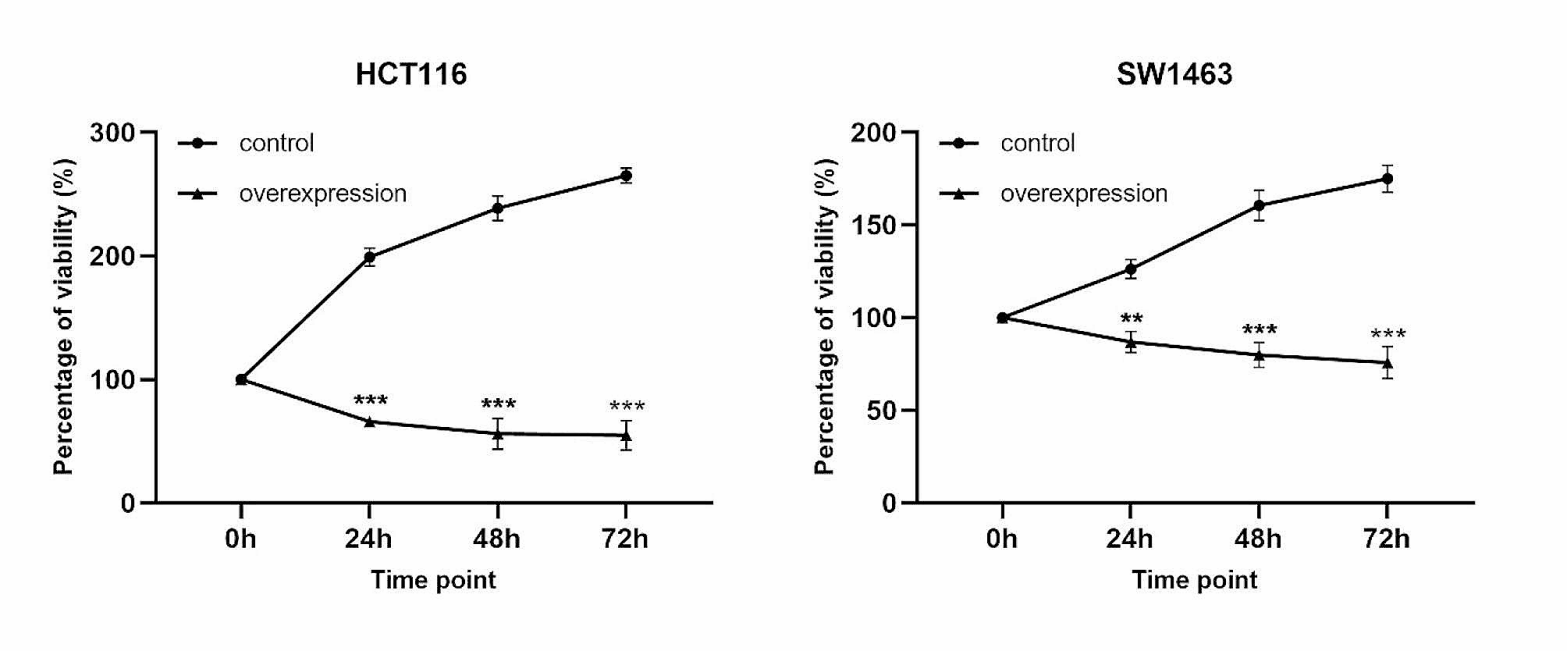
Overexpression of KCNMA1-AS2 inhibited proliferation of CRC cells. Cell proliferation was gauged by measuring the absorbance at 540 nm at 0, 24, 48, and 72 h in MTT assay. Cell viability percentages were calculated. KCNMA1-AS2 overexpression suppressed cell proliferation significantly across all time points in both HCT116 and SW1463 cells compared with the untransfected control (**p < 0.01, ***p < 0.001)

**Fig. 3:**
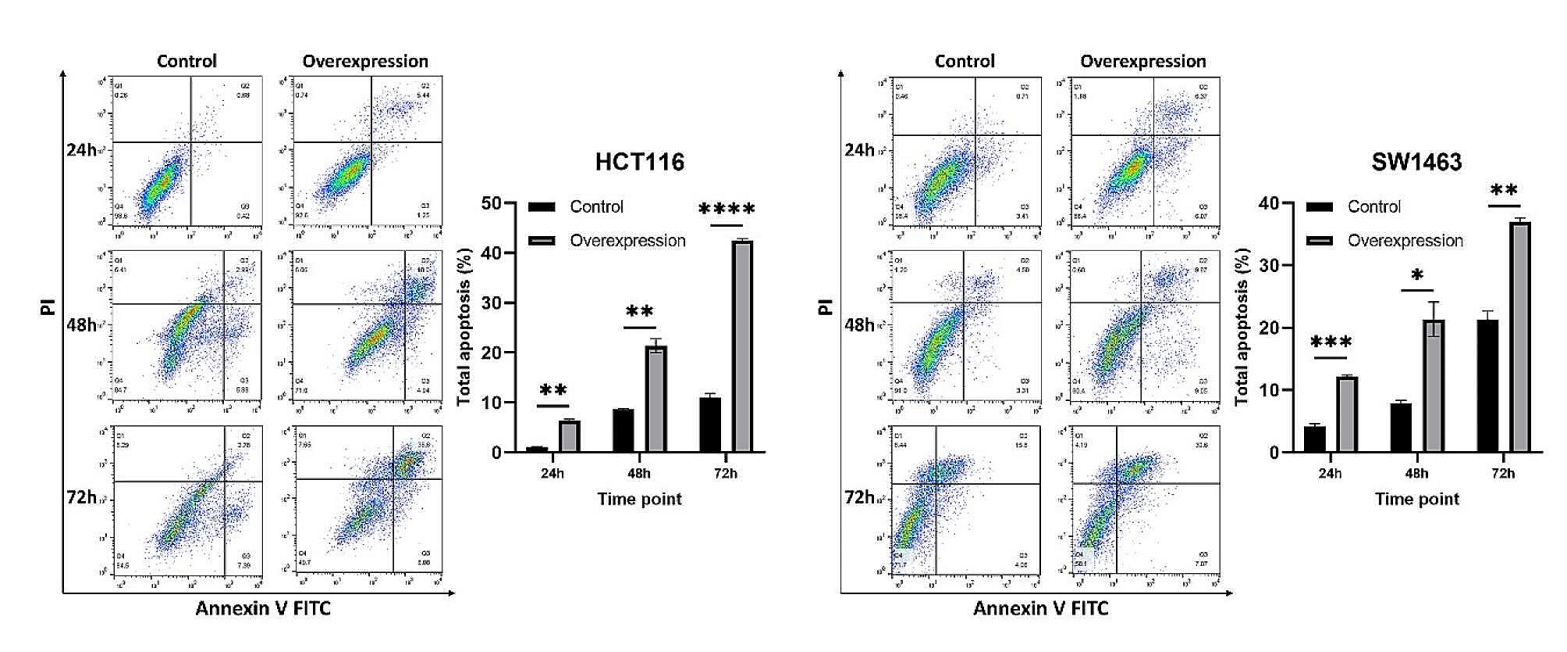
Overexpression of KCNMA1-AS2 promoted apoptosis of CRC cells. The effect of KCNMA1-AS2 overexpression on apoptosis of HCT116 and SW1463 cells examined by flow cytometry at 24, 48, and 72 h. A significant increase in apoptosis was detected in the overexpressed group compared to the untransfected controls at different time points (* *p* < 0.05, **p < 0.01, ***p < 0.001, ****p < 0.0001)

After inducing KCNMA1-AS2 overexpression in HCT116 and SW1463 cells, absorbance at 540 nm was measured at 0, 24, 48, and 72 h to determine cell viability percentages. For both cell lines, when KCNMA1-AS2 was overexpressed, a distinct decline in cell viability was evident compared to the untransfected control group at each corresponding time point (Fig. 2). Thus, it can be inferred that KCNMA1-AS2 overexpression inhibited CRC cell proliferation. The influence of KCNMA1-AS2 overexpression on apoptosis of CRC cells was shown in Fig. 3. Significantly augmented apoptosis was observed at each time point in the overexpressed group compared with the untransfected control group for both cell lines. These findings suggest that enhanced KCNMA1-AS2 expression may encourage apoptosis of CRC cells.

### Overexpression of KCNMA1-AS2 arrested cells at S phase and suppressed migration of CRC cells

**Fig. 4:**
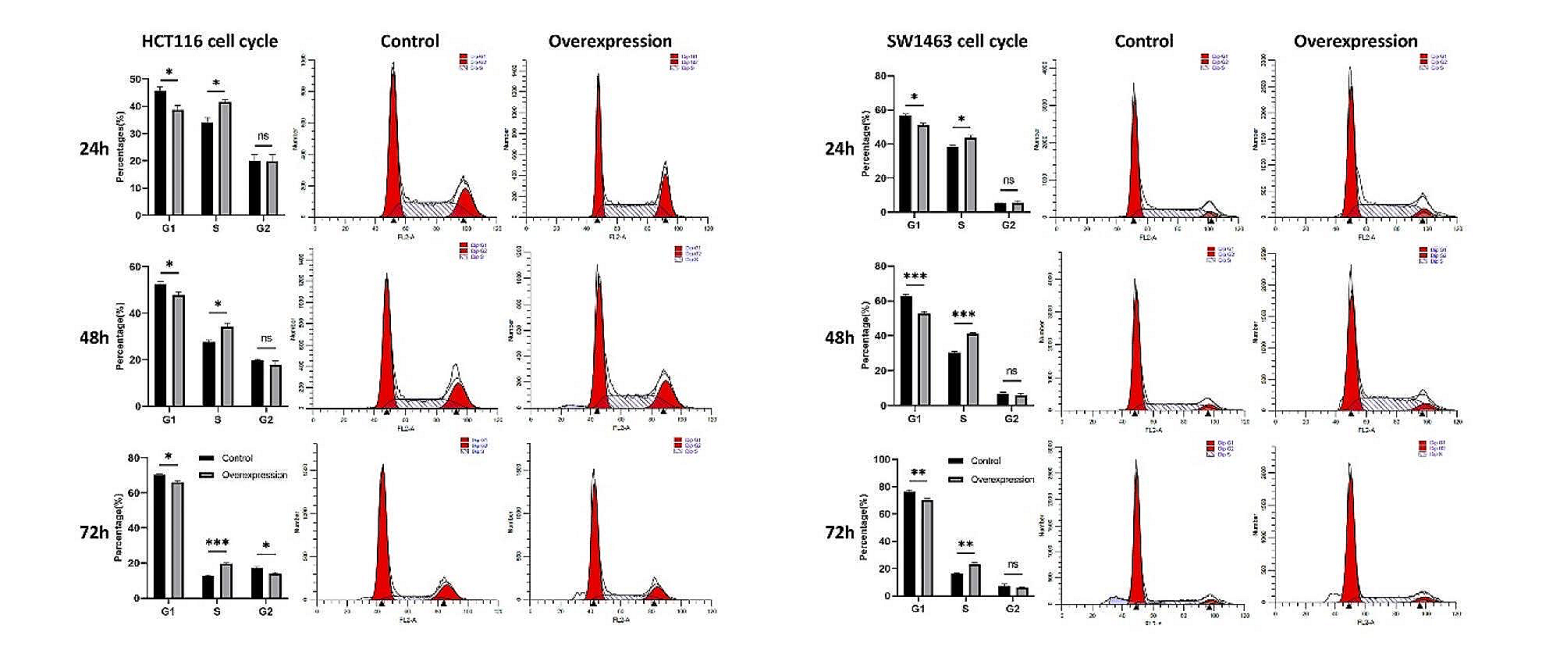
Overexpression of KCNMA1-AS2 arrested cells at S phase. The effects of lncRNA KCNMA1-AS2 overexpression on the cell cycle dynamics of HCT116 and SW1463 cells analyzed by flow cytometry at 24, 48, and 72 h. A significant shift towards S phase was observed in the cells overexpressing KCNMA1-AS2 compared to the untransfected controls. Concurrently, significant decrease in the percentage of G1 phase cells was observed (ns = not significant when *p* > 0.05, *p < 0.05, **p < 0.01, ***p < 0.001)

The effect of KCNMA1-AS2 overexpression on the cell cycle progression of HCT116 and SW1463 cells was illustrated in Fig. 4. Conspicuous accumulation of cells in S phase was found for both cell lines 24, 48, and 72 h post-transfection of KCNMA1-AS2, especially when compared with the untransfected controls. The findings also indicated a G1-to-S phase transition, leading to a halt or arrest at S phase for both cell lines. Nonetheless, no significant alteration was noted in the proportion of G2 phase cells until 72 h for HCT116 cell line. The actual difference was not so large between control and overexpression groups (17.1 ± 0.91% vs. 14.1 ± 0.42%) despite a statistically significant (*p < 0.05) difference was identified 72 h post-overexpression for HCT116 cell line.

**Fig. 5:**
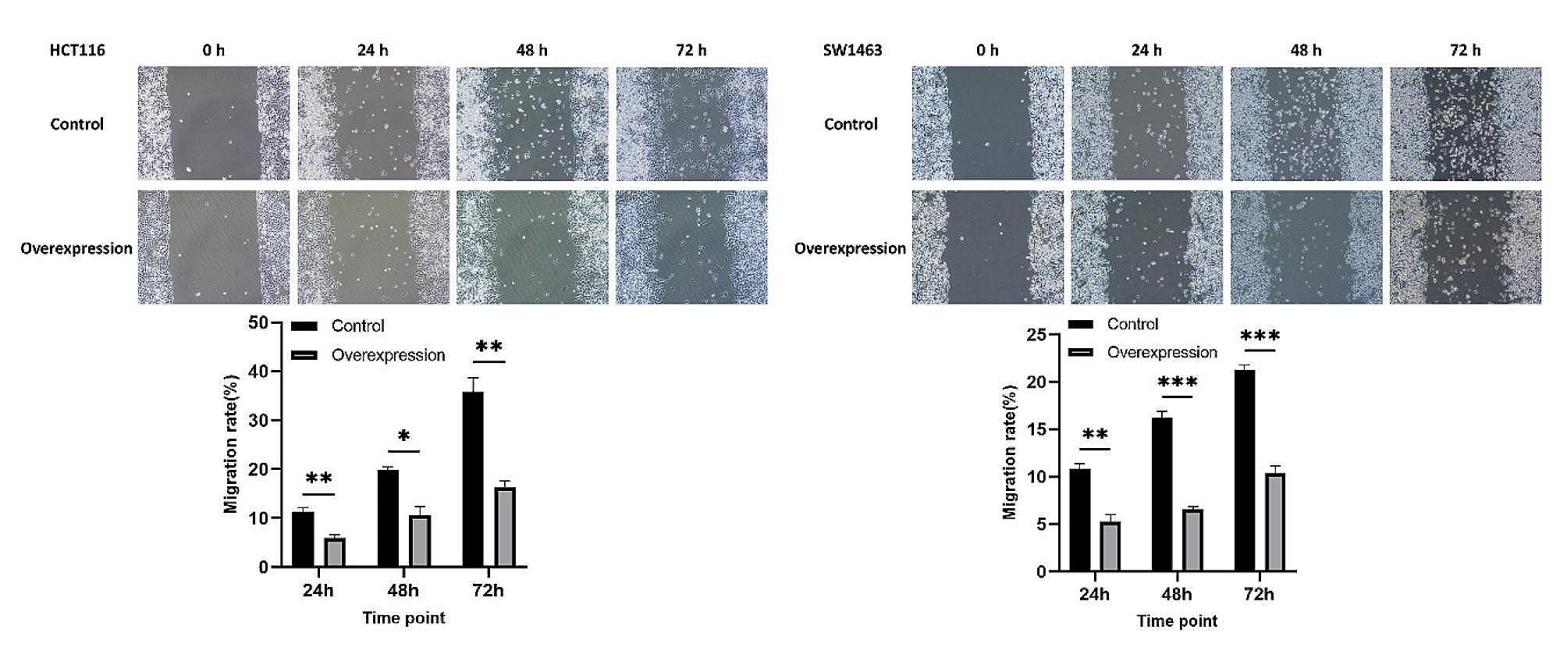
Overexpression of KCNMA1-AS2 suppressed migration of CRC cells. Scratch wound healing assay was conducted to evaluate the migration patterns of HCT116 and SW1463 cells 24, 48, and 72 h following KCNMA1-AS2 overexpression. Cells moved towards the wound center over time. However, the cell mobility was reduced in the overexpression group compared to the untransfected controls. The CRC cells overexpressing KCNMA1-AS2 showed significant slower migration compared to the control at 24, 48, and 72 h post-transfection (*p < 0.05, **p < 0.01, ***p < 0.001)

In the wound healing assay to examine the effect of KCNMA1-AS2 overexpression on the migratory capability of CRC cells, wound width was recorded at 0, 24, 48, and 72 h (Fig. 5). A movement toward the center of the wound was observed over time for both cell lines. However, the untransfected control cells advanced more rapidly than the cells overexpressing KCNMA1-AS2. Notably, CRC cells overexpressing KCNMA1-AS2 exhibited significantly slower migration rates at 24, 48, and 72 h compared to the controls. Thus, KCNMA1-AS2 overexpression effectively curbed the migration of CRC cells.

### KCNMA1-AS2 as a sponge for miR-1227-5p, with MTHFR and ST8SIA2 as potential targets of miR-1227-5p, specifically in relation to CRC

**Fig. 6:**
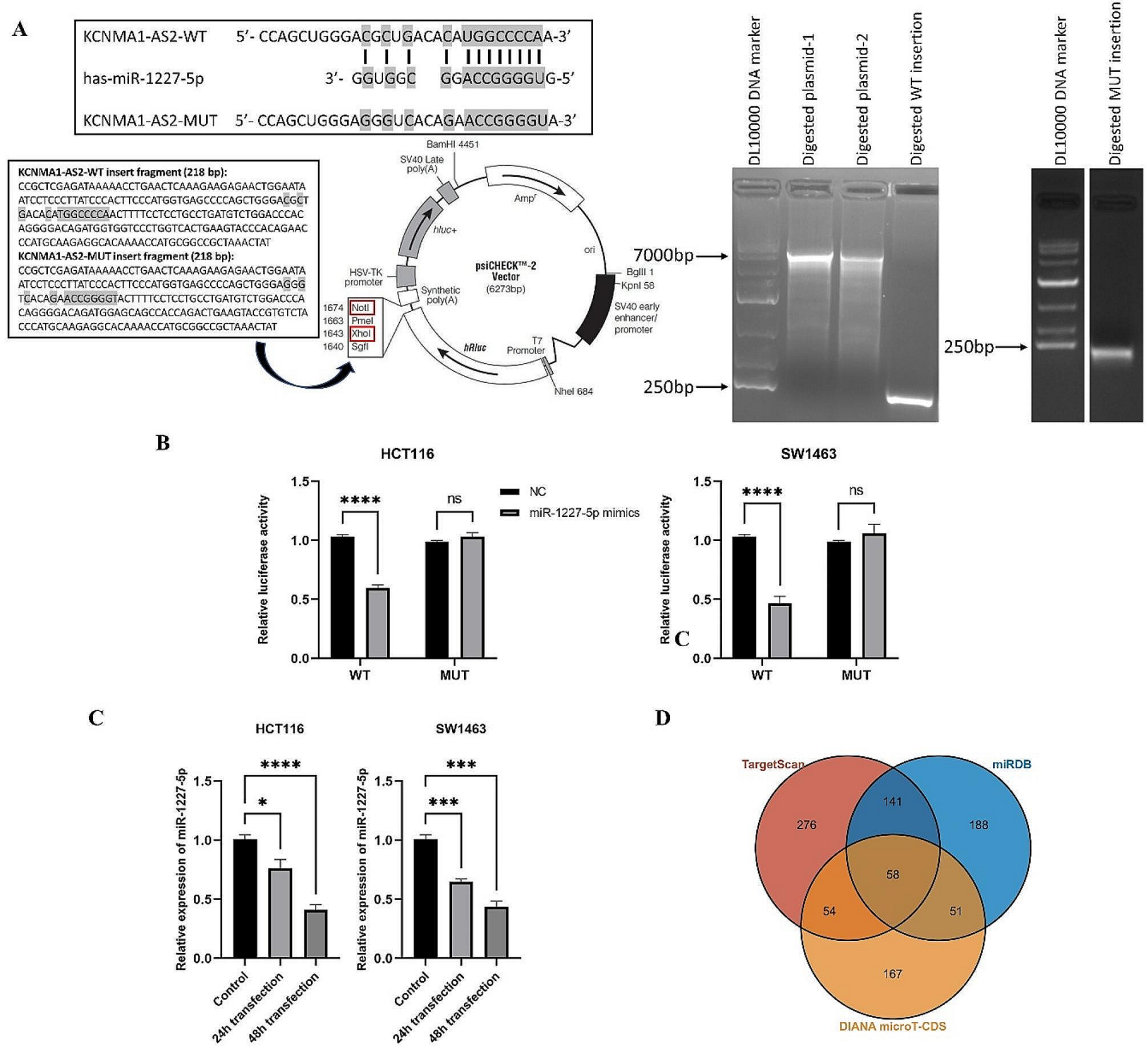
KCNMA1-AS2 as a sponge for miR-1227-5p and the prediction of target genes of miR-1227-5p. (**A**) Predicted interaction sites between miR-1227-5p and lncRNA KCNMA1-AS2 and the psiCHECK-2 plasmid and KCNMA1-AS2 WT/MUT insert fragments underwent digestion with the XhoI/NotI restriction enzyme. The psiCHECK-2 plasmid functioned as the base vector. KCNMA1-AS2 wild-type (WT) or mutant (MUT) fragments and psiCHECK-2 plasmid were digested via the XhoI/NotI restriction enzyme, leading to the formation of the recombinant plasmids by ligation. (**B**) The relative luciferase activities were quantified 48 h after co-transfection in HCT116 and SW1463 cells using psiCHECK-2 KCNMA1-AS2 WT/MUT and miR-1227-5p mimics or control. Luciferase activity was significantly reduced in the presence of miR-1227-5p mimics for KCNMA1-AS2 WT but remained unaffected for the MUT. (**C**) qPCR was used to determine the relative expression levels of miR-1227-5p in HCT116 and SW1463 cells 24 h and 48 h after KCNMA1-AS2 transfection. qPCR indicated significantly reduced expression 24 h and 48 h post-overexpression in both cell lines, supporting the interaction between KCNMA1-AS2 and miR-1227-5p in CRC cells (NC: negative control, ns = not significant when *p* > 0.05, * *p* < 0.05, ***p < 0.001, ****p < 0.0001). (**D**) The Venn diagram showcased target genes as follows: 529 predicted by TargetScan, 438 by miRDB, and 330 by DIANA microT-CDS. Overlaps between every pair of databases were also highlighted. Notably, 58 genes were mutually identified by all three databases after applying specific filtering criteria: TargetScan (Cumulative weighted context + + score ≤ -0.3), miRDB (score ≥ 60), and DIANA microT-CDS (miTG score ≥ 0.8)

DIANA tools lncBase v.2 revealed that miR-1227-5p was a potential target for lncRNA KCNMA1-AS2 with an overall score 0.969 and it had been reported to play an important role in lung cancer and osteosarcoma [21, 22]. To generate KCNMA1-AS2 WT/MUT plasmids, the psiCHECK-2 vector’s NotI and XhoI sites were employed as insertion restriction enzyme sites. Both the insert fragment and the empty psiCHECK-2 vector underwent restriction with NotI and XhoI, followed by gel electrophoresis as illustrated in Fig. 6A. Following ligation, transformation, cloning, and plasmid extraction, the reconstructed plasmid was sent to Sangon Biotech Co., Ltd. for sequencing confirmation. The reconstructed plasmid, thus prepared, was ready for use in subsequent luciferase reporter assays. The miR-1227-5p mimics significantly diminished the luciferase activity of KCNMA1-AS2 WT but had no effect on KCNMA1-AS2 MUT in both cell lines (Fig. 6B). This finding implied a binding interaction between KCNMA1-AS2 and miR-1227-5p. Additionally, qPCR analyses showcased that lncRNA KCNMA1-AS2 overexpression was associated with significantly reduced expression of miR-1227-5p at both 24 h and 48 h post-transfection for both HCT116 and SW1463 cell lines (Fig. 6C). This finding further supported the interaction between lncRNA KCNMA1-AS2 and miR-1227-5p within CRC cells.

From the original predictions, miRDB identified 744 genes, TargetScan highlighted 3,880 genes, and DIANA microT-CDS pinpointed 734 genes. After applying specific selection criteria, the counts were refined to 438, 529, and 330 genes for each database, respectively. A subset of 58 genes was consistently recognized by all the three databases (Fig. 6D). Ten cancer-associated genes were identified in the bioinformatic analysis of these 58 target genes by the KOBAS tool (Table S3). Of particular significance, MTHFR and ST8SIA2 were linked with CRC and will be prioritized in subsequent research. The other cancer-associated genes could also provide promising leads for future investigation.

## Discussion

This study has demonstrated for the first time that lncRNA KCNMA1-AS2 may function as a tumor suppressor in the evolution and progression of CRC. This research provides initial evidence that lncRNA KCNMA1-AS2 was undetectable in tumor tissue samples, as indicated by its negative infinite log_2_ (Fold Change) in sequencing results. Further validation in CRC tissue samples and cell lines revealed a pronounced decrease in KCNMA1-AS2 expression in tumorous tissues across all the cancer specimens from 10 patients compared to non-tumorous samples. This trend of reduced expression was mirrored in all CRC cell lines when compared with NCM460. Motivated by its attenuated expression in CRC, we aimed to elevate KCNMA1-AS2 levels to discern its effects on CRC cellular processes. Subsequent overexpression of KCNMA1-AS2 in CRC cell lines resulted in a significant reduction in cell proliferation, heightened apoptosis rate, cell cycle arrest in the S phase, and significant curbing of CRC cell migration relative to control groups.

Extensive literature review suggests that lncRNAs function as tumor suppressors in CRC development and progression, affecting CRC cell behaviors. Our findings are supported by previous researches showing that overexpressing certain suppressed lncRNAs inhibits CRC cell growth, promotes apoptosis, and diminishes migration. For instance, lncRNA-BCAT1 is significantly decreased in HCT116 and SW480 cells, implying its tumor-suppressing role in CRC, while overexpression of BCAT1 may lead to lower proliferation [23]. Another lncRNA, MEF2C-AS1, when overexpressed, significantly reduces CRC cell growth and migration [24]. Similarly, lncRNA KIAA0125, as a tumor suppressor, curtails CRC cell growth, migration, and invasion via the Wnt/β-catenin pathway [25]. In this study, enhanced lncRNA KCNMA1-AS2 expression led to S phase arrest in the cell cycle, a finding echoed to other research. For instance, lncRNA RPLP0P2 knockdown showed an accumulation of RKO cells in S phase, but fewer cells in G1 phase, suggesting S-to-G2/M phase transition arrest [26]. Similarly, upregulated lncRNA SNHG4 in CRC arrests colorectal cells in S phase, possibly interacting with CDK1 to boost CRC cell proliferation [27]. In contrast, other lncRNAs, like lncRNA-BCAT1, induce G1 phase arrest [23], while NR2F2-AS1 siRNA acts via Cyclin D1 downregulation, causing G0/G1 phase arrest [28]. Previous researches have characterized the fundamental mechanisms by which tumor suppressor lncRNAs influence proliferation, apoptosis, cell cycle migration, and invasion by altering specific related genes or biological pathways. For example, LINC00152 overexpression was associated with significant increase of proliferation-associated molecule Ki-67 and apoptosis-linked molecules like Bcl-2, and heightened levels of Fas in HT29 and SW480 cells [29]. The reduced expression of lncRNA GAS5 in CRC tissues and various CRC cell lines affects apoptosis of CRC by interacting with miR‑182‑5p/FOXO3a axis, where FOXO3a promotes apoptosis by inducing FasL and TRAIL ligand expression [30]. Understanding the underlying mechanisms of KCNMA1-AS2 is crucial for clarifying CRC gene regulation.

The role of KCNMA1-AS2 as a sponge for miR-1227-5p was confirmed through dual luciferase reporter assays and qPCR analyses in this study. A study noted the downregulation of miR-1227-5p in gastric cancer [31]. and another study linked its expression levels to cigarette smoking in CRC [32]. A comprehensive study revealed miR-1227-5p as a potential target of lncRNA OR3A4 in osteosarcoma, the overexpression of which was linked to poor patient outcomes. OR3A4 was found to bolster osteosarcoma cell growth and invasion by targeting miR-1227-5p [22]. In another study, miR-1227-5p was associated with the histone chaperone Spt16 in lung cancer. Under-expression of miR-1227-5p suppressed cell growth and promoted apoptosis. Introducing a miR-1227-5p mimic reduced Spt16 expression, a finding supported by luciferase assays [21]. The research and publication surrounding miR-1227-5p have been notably limited, especially within the context of CRC. This scarcity highlights its potential as a promising target for addressing gaps in this field. Bioinformatic analysis based on lncRNA-miRNA-mRNA network identified 58 potential target genes, 10 of which are cancer-specific. Notably, MTHFR and ST8SIA2 are associated with CRC. MTHFR, linked with various diseases, has been shown to play a role in the growth and progression of colon neoplasms [33]. Studies have found a correlation between MTHFR polymorphisms and environmental factors such as air pollution [34, 35]. Previous research links methylation abnormalities in lncRNA H19 to MTHFR gene dysfunction in infertile men [36], while another study associates lncRNA HOTAIR with chemoresistance in esophageal cancer [37]. ST8SIA2, responsible for synthesizing polysialic acid, a molecule associated with metastasis [38], which is a potential molecular marker for metastatic neuroblastoma [39, 40]. Its role in cancer metastasis has been validated in studies, suggesting its significance in enhancing the invasion of non-small cell lung cancer cells [41]. Despite the known roles of MTHFR and ST8SIA2 in cancer, their links with lncRNAs are under-studied in CRC, indicating a promising avenue for future research. Metabolic pathways, as suggested by KOBAS analysis, could be a focus for characterizing the roles of these genes in CRC.

In light of these pivotal discoveries, lncRNA KCNMA1-AS2 may serve as a biomarker for early detection of CRC. The expression level of KCNMA1-AS2 in biological samples could potentially contribute to early detection of CRC in clinical practice.

However, there are some limitations to this study. The association between KCNMA1-AS2 and miR-1227-5p was not investigated in more patient samples due to some constraints. Additionally, the underlying mechanism of KCNMA1-AS2/miR-1227-5p interaction was not further clarified due to technical challenges. The complex interactions between lncRNA-miRNA-mRNA are central to deciphering cell regulation pathways. As such, the downstream genes, MTHFR and ST8SIA2, will be further investigated to decode the lncRNA-miRNA-mRNA network.

## Conclusions

The levels of lncRNA KCNMA1-AS2 expression were significantly decreased in both CRC tissue samples and cell lines compared to their non-cancerous counterparts. Overexpression of lncRNA KCNMA1-AS2 resulted in marked inhibition of cell proliferation and migration, increased cell apoptosis, and a higher proportion of cells arrested in the S phase. The binding of KCNMA1-AS2 to miR-1227-5p was confirmed, and subsequent bioinformatics analyses identified 58 potential downstream targets of miR-1227-5p across three databases. Among these, 10 were specifically related to cancer, and MTHFR and ST8SIA2 were predicted to be linked to CRC. This study introduces lncRNA KCNMA1-AS2 as a novel candidate with the potential to serve as a diagnostic and therapeutic biomarker for CRC.

### Electronic supplementary material

Below is the link to the electronic supplementary material.

Supplementary Material 1

## Data Availability

The sequence data that support the findings of this study have been deposited in National Center for Biotechnology Information (NCBI) and all data have been released. The datasets generated and/or analyzed during the current study are available in the NCBI repository. Accession link of SRA data: https://www.ncbi.nlm.nih.gov/sra/PRJNA1053608. BioSample accessions: SAMN38861695, SAMN38861696, SAMN38861697, SAMN38861698, SAMN38861699, SAMN38861700.

## Abbreviations

ANOVA: Analysis of variance
ceRNA: Competitive endogenous RNA
CRC: Colorectal cancer
lncRNA: Long noncoding RNA
miRNA: MicroRNA
MTT: 3-[4,5-dimethylthiazol-2-yl]-2,5 diphenyl tetrazolium bromide
MUT: Mutant
NC: Negative control
ncRNA: Non-coding RNA
PI: Propidium iodide
qPCR: Quantitative real-time polymerase chain reaction
WT: Wild-type
