# Supplementary material for: Long noncoding RNA KCNMA1-AS2 regulates the function of colorectal cancer cells and sponges miR-1227-5p
Source: BMC Cancer. 2024 Jul 18;24:857. doi: 10.1186/s12885-024-12608-9 (PMC11256649; doi:10.1186/s12885-024-12608-9)
Supplement: Supplementary file 1 — Supplementary Material 1 [file 12885_2024_12608_MOESM1_ESM.docx]

**
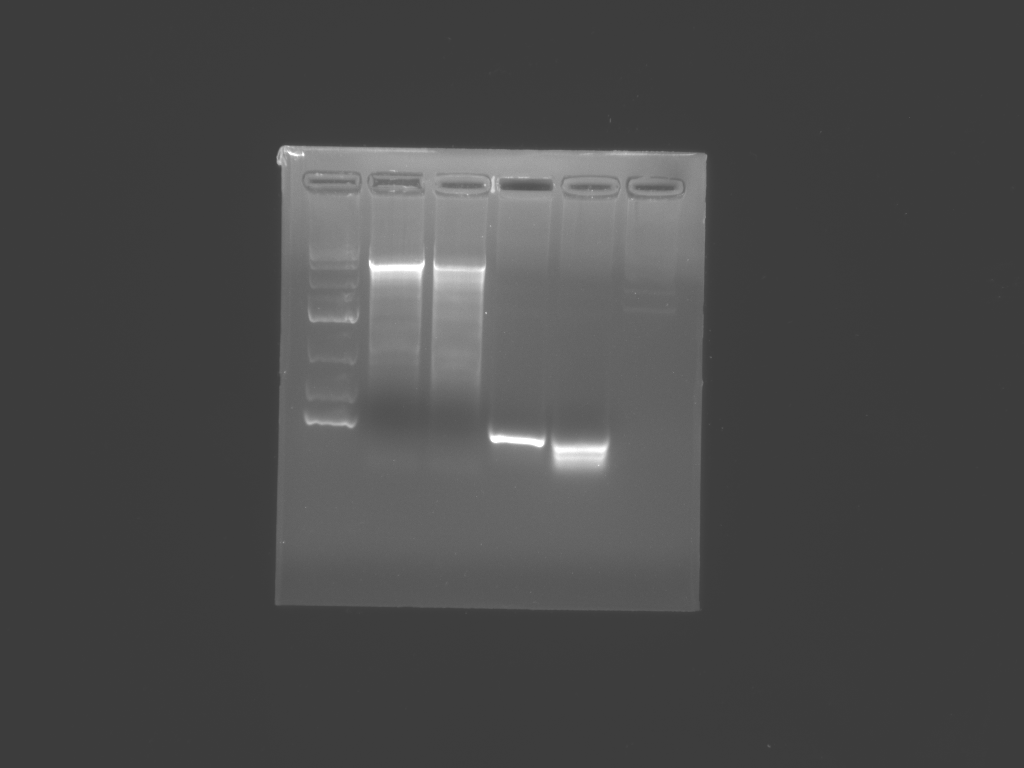
**

**Fig. S1.** Original gel image of psiCHECK-2 plasmid and KCNMA1-AS2 WT insert fragments underwent digestion with the XhoI/NotI restriction enzyme. Lane 1: DL10000 DNA marker, Lane 2: digested plasmid-1, Lane 3: digested plasmid-2, Lane 4: digested WT insertion.

**
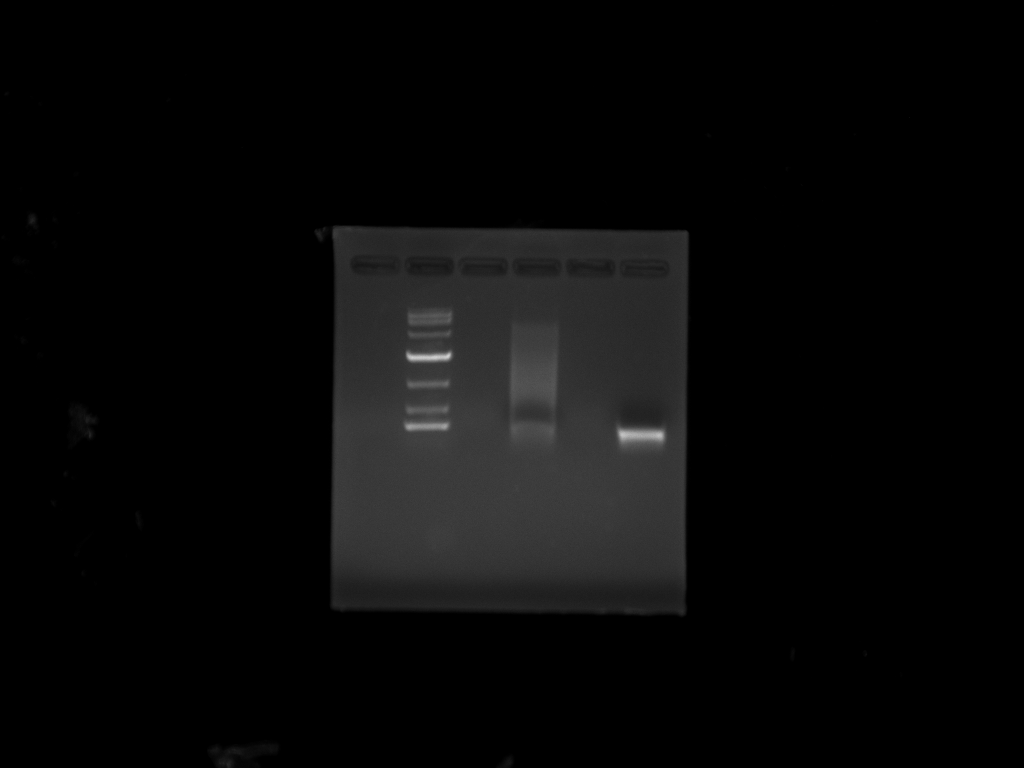
**

**Fig. S2.** Original gel image of KCNMA1-AS2 MUT insert fragments underwent digestion with the XhoI/NotI restriction enzyme. Lane 2: DL10000 DNA marker, Lane 6: digested MUT insertion.

**Table S1**. qPCR primers for GAPDH and KCNMA1-AS2.

| **Primers** | **Sequence (5’-3’)** |
| --- | --- |
| GAPDH Forward | ATTTGGCAGGTTTTTCTAGACG |
| GAPDH Reverse | ATCGTGGAAGGACTCATGACC |
| KCNMA1-AS2 Forward | CTCCCTTATCCCACTTCC |
| KCNMA1-AS2 Reverse | CTCTTGCATGGGTTCTGT |

**Table S2**. qPCR primers for U6 and miR-1227-5p.

| **Type** | **Primers** | **Sequence (5’-3’)** |
| --- | --- | --- |
| Reverse transcription primer | U6-A | AACGCTTCACGAATTTGCGT |
|  | miR-1227-5p-RT | CTCAACTGGTGTCGTGGAGTCGGCAATTCAGTTGAGCCACCGCC |
| U6 qPCR primer | U6-A | AACGCTTCACGAATTTGCGT |
|  | U6-S | CTCGCTTCGGCAGCACA |
| miR-1227-5p qPCR primer | universal primer A | TGGTGTCGTGGAGTCG |
|  | miR-1227-5p-S | ACACTCCAGCTGGGGTGGGGCCAGGCGGTGG |

**Table S3.** Bioinformatic analysis summary for ten cancer-related common predicted targets of miR-1227-5p.

| **Gene** | **Disease** | **Source** | **Pathway** | **Source** |
| --- | --- | --- | --- | --- |
| **ALOX5AP** | neuroblastoma | FunDO | Inflammation mediated by chemokine and cytokine signalling pathway | PANTHER |
|  |  |  | Fc epsilon RI signalling pathway | KEGG |
|  |  |  | Biosynthesis of specialised proresolving mediators (spms) | Reactome |
|  |  |  | Eicosanoid metabolism | BioCarta |
|  |  |  | Metabolism of lipids | Reactome |
|  |  |  | Arachidonic acid metabolism | Reactome |
|  |  |  | Synthesis of 5-eicosatetraenoic acids | Reactome |
|  |  |  | Metabolism | Reactome |
|  |  |  | Synthesis of Leukotrienes (LT) and Eoxins (EX) | Reactome |
|  |  |  | Fatty acid metabolism | Reactome |
|  |  |  | Synthesis of Lipoxins (LX) | Reactome |
| **ASIC1** | adenoid cystic cancer | FunDO | Inflammatory mediator regulation of TRP channels | KEGG |
|  |  |  | Transport of small molecules | Reactome |
|  |  |  | Stimuli-sensing channels | Reactome |
|  |  |  | Ion channel transport | Reactome |
| **GNAI2** | cancers | KEGG | S1P5 pathway | PID |
|  | cancers of endocrine organs | KEGG | Muscarinic acetylcholine receptor 2 and 4 signalling pathway | PANTHER |
|  | adrenal carcinoma | KEGG | Inflammation mediated by chemokine and cytokine signalling pathway | PANTHER |
|  |  |  | Opioid prodynorphin pathway | PANTHER |
|  |  |  | Estrogen signalling pathway | KEGG |
|  |  |  | Serotonergic synapse | KEGG |
|  |  |  | Opioid proopiomelanocortin pathway | PANTHER |
|  |  |  | PAR1-mediated thrombin signalling events | PID |
|  |  |  | Regulation of insulin secretion | Reactome |
|  |  |  | LPA receptor mediated events | PID |
|  |  |  | CXCR4-mediated signalling events | PID |
|  |  |  | Platelet activation | KEGG |
|  |  |  | Glutamatergic synapse | KEGG |
|  |  |  | Long-term depression | KEGG |
|  |  |  | Oxytocin signalling pathway | KEGG |
|  |  |  | Opioid proenkephalin pathway | PANTHER |
|  |  |  | Metabotropic glutamate receptor group III pathway | PANTHER |
|  |  |  | Chemokine signalling pathway | KEGG |
|  |  |  | IL8- and CXCR1-mediated signalling events | PID |
|  |  |  | 5HT1 type receptor mediated signalling pathway | PANTHER |
|  |  |  | Human cytomegalovirus infection | KEGG |
|  |  |  | Camp signalling pathway | KEGG |
|  |  |  | S1P1 pathway | PID |
|  |  |  | Axon guidance | KEGG |
|  |  |  | Leukocyte transendothelial migration | KEGG |
|  |  |  | Thromboxane A2 receptor signalling | PID |
|  |  |  | S1P3 pathway | PID |
|  |  |  | Heterotrimeric G-protein signalling pathway-Gi alpha and Gs alpha mediated pathway | PANTHER |
|  |  |  | PI3 kinase pathway | PANTHER |
|  |  |  | CXCR3-mediated signalling events | PID |
|  |  |  | S1P4 pathway | PID |
|  |  |  | Dopamine receptor mediated signalling pathway | PANTHER |
|  |  |  | Sphingolipid signalling pathway | KEGG |
|  |  |  | GPCR downstream signalling | Reactome |
|  |  |  | Retrograde endocannabinoid signalling | KEGG |
|  |  |  | Parkinson disease | KEGG |
|  |  |  | Plasma membrane estrogen receptor signalling | PID |
|  |  |  | Progesterone-mediated oocyte maturation | KEGG |
|  |  |  | Enkephalin release | PANTHER |
|  |  |  | Pathways in cancer | KEGG |
|  |  |  | Nongenotropic Androgen signalling | PID |
|  |  |  | Gastric acid secretion | KEGG |
|  |  |  | Human immunodeficiency virus 1 infection | KEGG |
|  |  |  | Metabotropic glutamate receptor group II pathway | PANTHER |
|  |  |  | Hedgehog signalling events mediated by Gli proteins | PID |
|  |  |  | Sphingosine 1-phosphate (S1P) pathway | PID |
|  |  |  | Cgmp-PKG signalling pathway | KEGG |
|  |  |  | Apelin signalling pathway | KEGG |
|  |  |  | Parathyroid hormone synthesis, secretion and action | KEGG |
|  |  |  | Cushing syndrome | KEGG |
|  |  |  | Rap1 signalling pathway | KEGG |
|  |  |  | Endothelins | PID |
|  |  |  | IL8- and CXCR2-mediated signalling events | PID |
|  |  |  | Alcoholism | KEGG |
|  |  |  | Renin secretion | KEGG |
|  |  |  | Morphine addiction | KEGG |
|  |  |  | Cholinergic synapse | KEGG |
|  |  |  | Gap junction | KEGG |
|  |  |  | Circadian entrainment | KEGG |
|  |  |  | Gabaergic synapse | KEGG |
|  |  |  | Dopaminergic synapse | KEGG |
|  |  |  | Melanogenesis | KEGG |
|  |  |  | S1P2 pathway | PID |
|  |  |  | Cocaine addiction | KEGG |
|  |  |  | Adrenergic signalling in cardiomyocytes | KEGG |
|  |  |  | Pertussis | KEGG |
|  |  |  | Relaxin signalling pathway | KEGG |
|  |  |  | Chagas disease (American trypanosomiasis) | KEGG |
|  |  |  | Adrenaline, noradrenaline inhibits insulin secretion | Reactome |
|  |  |  | Regulation of lipolysis in adipocytes | KEGG |
|  |  |  | Toxoplasmosis | KEGG |
|  |  |  | Signal amplification | Reactome |
|  |  |  | Cooperation of PDCL (phlp1) and tric/CCT in G-protein beta folding | Reactome |
|  |  |  | ESR-mediated signalling | Reactome |
|  |  |  | Neuronal system | Reactome |
|  |  |  | Opioid signalling | Reactome |
|  |  |  | Signalling by GPCR | Reactome |
|  |  |  | GABA receptor activation | Reactome |
|  |  |  | Integration of energy metabolism | Reactome |
|  |  |  | Metabolism of proteins | Reactome |
|  |  |  | Metabolism | Reactome |
|  |  |  | G alpha (z) signalling events | Reactome |
|  |  |  | Signalling by Nuclear Receptors | Reactome |
|  |  |  | Signal transduction | Reactome |
|  |  |  | ADP signalling through P2Y purinoceptor 12 | Reactome |
|  |  |  | G alpha (s) signalling events | Reactome |
|  |  |  | PLC beta mediated events | Reactome |
|  |  |  | Haemostasis | Reactome |
|  |  |  | Adenylate cyclase inhibitory pathway | Reactome |
|  |  |  | G-protein activation | Reactome |
|  |  |  | G alpha (i) signalling events | Reactome |
|  |  |  | Protein folding | Reactome |
|  |  |  | Platelet activation, signalling and aggregation | Reactome |
|  |  |  | Transmission across Chemical Synapses | Reactome |
|  |  |  | Extra-nuclear estrogen signalling | Reactome |
|  |  |  | Chaperonin-mediated protein folding | Reactome |
|  |  |  | Neurotransmitter receptors and postsynaptic signal transmission | Reactome |
|  |  |  | G-protein mediated events | Reactome |
|  |  |  | Activation of GABAB receptors | Reactome |
|  |  |  | GABA B receptor activation | Reactome |
| **IGF2BP1** | prostate cancer | NHGRI GWAS Catalog | Regulation of nuclear beta catenin signalling and target gene transcription | PID |
|  | cancer | FunDO | Micrornas in cancer | KEGG |
|  |  |  | MAPK6/MAPK4 signalling | Reactome |
|  |  |  | Signal transduction | Reactome |
|  |  |  | Insulin-like Growth Factor-2 mrna Binding Proteins (IGF2BPs/imps/vickzs) bind RNA | Reactome |
|  |  |  | MAPK family signalling cascades | Reactome |
|  |  |  | Metabolism of RNA | Reactome |
| **MAPK8IP1** | testicular tumour | FunDO | MAPK signalling pathway | KEGG |
|  | prostate cancer | FunDO | Reelin signalling pathway | PID |
| **MERTK** | embryoma | FunDO | Haemostasis | Reactome |
|  |  |  | Cell surface interactions at the vascular wall | Reactome |
| **MTHFR** | cancer | GAD | Metabolic pathways | KEGG |
|  | esophageal cancer | GAD | Carbon metabolism | KEGG |
|  | liver cancer | GAD | One carbon pool by folate | KEGG |
|  | lung cancer | GAD | Folate transformations I | BioCyc |
|  | breast cancer | GAD | Antifolate resistance | KEGG |
|  | stomach cancer | GAD | Metabolism of folate and pterines | Reactome |
|  | bladder cancer | GAD | Metabolism of water-soluble vitamins and cofactors | Reactome |
|  | **colorectal cancer** | GAD | Metabolism | Reactome |
|  | cancer | FunDO | Metabolism of vitamins and cofactors | Reactome |
|  | gastric cardia cancer | GAD |  |  |
|  | esophageal neoplasms | GAD |  |  |
|  | adenocarcinoma | GAD |  |  |
|  | stomach neoplasms | GAD |  |  |
|  | pancreatic neoplasms | GAD |  |  |
|  | ovarian cancer- methotrexate related toxicity | GAD |  |  |
|  | gastrointestinal neoplasms | GAD |  |  |
|  | oesophageal neoplasm | GAD |  |  |
|  | pancreatic neoplasm | GAD |  |  |
| **PAX5** | cancers | KEGG | Transcriptional misregulation in cancer | KEGG |
|  | cancer | GAD | C-MYB transcription factor network | PID |
|  | cancers of haematopoietic and lymphoid tissues | KEGG | RUNX1 regulates transcription of genes involved in BCR signalling | Reactome |
|  | lymphoplasmacytic lymphoma | KEGG | Gene expression (Transcription) | Reactome |
|  | cancer | FunDO | Transcriptional regulation by RUNX1 | Reactome |
|  | precursor b-cell lymphoblastic leukemia-lymphoma | GAD | Generic transcription pathway | Reactome |
|  |  |  | Rna polymerase ii transcription | Reactome |
| **ST8SIA2** | **colorectal cancer (diet interaction)** | NHGRI GWAS Catalog | NCAM signalling for neurite out-growth | Reactome |
|  |  |  | Biosynthesis of ABH and Lewis epitopes from type 2 precursor disaccharide | BioCyc |
|  |  |  | Asparagine N-linked glycosylation | Reactome |
|  |  |  | Neolacto-series glycosphingolipids biosynthesis | BioCyc |
|  |  |  | Super pathway of glycosphingolipids biosynthesis | BioCyc |
|  |  |  | Terminal O-glycans residues modification | BioCyc |
|  |  |  | Developmental biology | Reactome |
|  |  |  | Synthesis of substrates in N-glycan biosythesis | Reactome |
|  |  |  | Axon guidance | Reactome |
|  |  |  | Metabolism of proteins | Reactome |
|  |  |  | Post-translational protein modification | Reactome |
|  |  |  | N-Glycan antennae elongation | Reactome |
|  |  |  | Sialic acid metabolism | Reactome |
|  |  |  | N-glycan antennae elongation in the medial/trans-Golgi | Reactome |
|  |  |  | NCAM1 interactions | Reactome |
|  |  |  | Transport to the Golgi and subsequent modification | Reactome |
|  |  |  | Biosynthesis of the N-glycan precursor (dolichol lipid-linked oligosaccharide, LLO) and transfer to a nascent protein | Reactome |
| **TFE3** | cancers | KEGG | Transcriptional misregulation in cancer | KEGG |
|  | cancers of soft tissues and bone | KEGG | Renal cell carcinoma | KEGG |
|  | alveolar soft part sarcoma | KEGG | Regulation of nuclear SMAD2/3 signalling | PID |
|  | cancers of the urinary system | KEGG | E2F transcription factor network | PID |
|  | renal cell carcinoma | KEGG | Mitophagy - animal | KEGG |
|  | Renal cell carcinoma, papillary, 1 | OMIM |  |  |

**FunDO**: Functional disease ontology annotation database;**PANTHER**:protein analysis through evolutionary relationships database;**KEGG**: Kyoto Encyclopedia of Genes and Genomes;**Reactome**:Reactome Pathway Database;**BioCarta**: BioCarta pathway database;**PID**:Pathway Interaction Database;**NHGRI** **GWAS** **Catalog**: National Human Genome Research Institute Home genome-wide association studies database;**GAD**:Genetic Association Database;**BioCyc**: BioCyc genome database collection;**OMIM**: Online Mendelian Inheritance in Mandatabase.
